# Supplementary material for: Nutrition Education Programs for Adults with Neurological Diseases Are Lacking: A Scoping Review
Source: Nutrients. 2022 Apr 10;14(8):1577. doi: 10.3390/nu14081577 (PMC9030740; doi:10.3390/nu14081577)
Supplement: Supplementary file 1 [file nutrients-14-01577-s001.zip › Scoping review_Nutrition education neurol_table S2.pdf]

Nutrition education programs for adults with neurological diseases are lacking: a scoping review

Table S2. Search strategy for MEDLINE (Ovid) and CINAHL databases. January 2022.

MEDLINE (Ovid)

**No.**	Search terms	Retrieved
1	exp "autoimmune diseases of the nervous system"/ or exp autonomic nervous system diseases/ or exp central nervous system diseases/ or exp cranial nerve diseases/ or exp demyelinating diseases/ or exp neurodegenerative diseases/ or exp neuromuscular diseases/	1971299
2	Multiple sclerosis.mp. or Parkinson's.mp. or Huntington's.mp. or motor neurone.mp. or Alzheimer's.mp. or dementia.mp. or amyotrophic lateral sclerosis.mp. or stroke.mp. or epilepsy.mp.	924146
3	1 or 2	2279571
4	exp Diet/ or diet*.ti,ab.	735446
5	exp Nutrition Therapy/ or nutrition*.ti,ab.	378612
6	exp Diet Therapy/	59231
7	(diet* adj2 (behavio?r* or quality or pattern* or change)).ab.	26866
8	4 or 5 or 6 or 7	968556
9	exp Health Education/	256186
10	exp Patient Education as Topic/ or patient education.ti,ab.	99169
11	education* program*.ti,ab.	46469
12	exp Health Promotion/	82293
13	diet* education.ti,ab.	682
14	nutrition* education.ti,ab.	6001
15	9 or 10 or 11 or 12 or 13 or 14	305150
16	3 and 8 and 15	773
17	limit 16 to (English language and humans)	688

CINAHL

**No.**	Search terms	Retrieved
S1	(MH "Nervous System Diseases") OR (MH "Autoimmune Diseases of the Nervous System+") OR (MH "Autonomic Nervous System Diseases+") OR (MH "Central Nervous System Diseases+") OR (MH "Cranial Nerve Diseases+") OR (MH "Demyelinating Diseases+") OR (MH "Neurodegenerative Diseases+") OR (MH "Parkinsonian Disorders+") OR (MH "Neuromuscular Diseases+")	503637
S2	AB Multiple sclerosis or Parkinson's or Huntington's or motor neurone or Alzheimer's or dementia or prion or amyotrophic lateral sclerosis or stroke or epilepsy	187517
S3	1 or 2	559013
S4	AB diet*	117813
S5	(MH "Diet+")	133347
S6	(MH "Diet Therapy+")	35430
S7	AB "diet* therapy" or "nutrition therapy"	1340
S8	AB "diet* behavio?r*" or "diet* quality" or "diet* pattern*" or "diet* change"	9230
S9	S4 or S5 or S6 or S7 or S8	207,624
S10	(MH "Health Education+")	137306
S11	(MH "Patient Education+")	83196
S12	(MH "Health Promotion+")	76111
S13	AB "diet* education"	314
S14	AB "education* program*"	26054

S15	AB "nutrition* education"	3073
S16	S10 or S11 or S12 or S13 or S14 or S15	223,079
S17	S3 and S9 and S16	522
