# Supplementary material for: Nutrition Education Programs for Adults with Neurological Diseases Are Lacking: A Scoping Review
Source: Nutrients. 2022 Apr 10;14(8):1577. doi: 10.3390/nu14081577 (PMC9030740; doi:10.3390/nu14081577)
Supplement: Supplementary file 1 [file nutrients-14-01577-s001.zip › Scoping review_Nutrition education neurol_table S3.pdf]

Nutrition education programs for adults with neurological diseases are lacking: a scoping review

Table S3. Neurological disease organizations contacted by email.

Neurological disease	Organization name	Web address	Response received (yes/no)
Dementia	Dementia Australia	https://www.dementia.org.au/	Yes
	Alzheimer's WA	https://www.alzheimerswa.org.au/	Yes
	Alzheimer's Association	https://www.alz.org/	Yes
	Alzheimer's Disease International	https://www.alz.co.uk/	Yes
	Alzheimer's Association Australia	https://www.alz.org/au/dementia-alzheimers-australia.asp	No
	Alzheimer's Queensland	https://www.alzheimersonline.org/	No
	Alzheimer's Society	https://www.alzheimers.org.uk/	No
	Alzheimer's Foundation of America	https://alzfdn.org/	No
Epilepsy	Epilepsy Action Australia	https://www.epilepsy.org.au/	Yes
	Epilepsy Society	https://www.epilepsysociety.org.uk/	Yes
	Epilepsy Foundation END EPILEPSY	https://www.epilepsy.com/	Yes
	American Epilepsy Society	http://www.aesnet.org/	Yes
	Epilepsy Foundation	http://epilepsyfoundation.org.au/	No
	International League Against Epilepsy and International Bureau for Epilepsy	https://www.epilepsy.org/	No
	Epilepsy Society	http://www.epilepsysociety.org.uk/	No
	The Epilepsy Centre	https://epilepsycentre.org.au/	No
	Epilepsy Foundation	https://epilepsyfoundation.org/	No
	Epilepsy Australia	http://www.epilepsyaustralia.net/	No
	International League Against Epilepsy	https://www.ilae.org/	No
Huntington's disease	Huntington's Disease Society of America	https://hdsa.org/	Yes
	Huntington's NSW ACT	https://www.huntingtonsnsw.org.au/	Yes
	Huntington's Disease Association	https://www.hda.org.uk/	Yes
	Huntington Society of Canada	https://www.huntingtonsociety.ca/	Yes
	Huntington's Western Australia	https://www.huntingtonswa.org.au/	Yes
	The International Huntington Association	https://huntington-disease.org/	No
	Huntingtons Queensland	https://huntingtonsqld.org.au/	No
Motor neurone disease (MND)	Motor Neurone Disease Association	https://www.mndassociation.org/	Yes
	A Life Story Foundation	https://www.alifestoryfoundation.org/	Yes
	MND Australia	https://www.mndaust.asn.au/Home	Yes
	MND Victoria	https://www.mnd.asn.au/	Yes
	International Alliance of ALS/MND Associations	https://www.alsmndalliance.org/	Yes
	MND NSW	https://www.mndnsw.asn.au/	No
Multiple sclerosis (MS)	MS	https://www.ms.org.au/	Yes
	MS Research Australia	https://msra.org.au/	Yes
	National Multiple Sclerosis Society	https://www.nationalmssociety.org/	Yes
	Multiple Sclerosis New Zealand	https://www.msnz.org.nz/	Yes
	The MS Society	https://mssociety.ca/	Yes
	MS Australia	https://www.msaustralia.org.au/	Yes
	MSWA	https://www.mswa.org.au	Yes

	The Multiple Sclerosis Society of South Australia and Northern Territory	https://www.ms.asn.au/	Yes
	MS Society	https://www.mssociety.org.uk/	Yes
	MS International Federation	https://www.msif.org/	No
Parkinson's disease	Parkinson's Foundation	https://www.parkinson.org/	Yes
	International Parkinson and Movement Disorder Society	https://www.movementdisorders.org/MDS.htm	Yes
	American Parkinson Disease Association	https://www.apdaparkinson.org/	Yes
	European Parkinson's Disease Association	https://www.epda.eu.com/	Yes
	Parkinson's NSW	https://www.parkinsonsnsw.org.au/	Yes
	The Michael J. Fox Foundation	https://www.michaeljfox.org/	No
	Parkinson's Australia	https://www.parkinsons.org.au/	No
	ParkinsonNet	https://www.parkinsonnet.com/	No
	NeuroWellness	https://www.parkinsonsinternational.com/	No
Stroke	American Stroke Association	https://www.stroke.org/	Yes
	Stroke Foundation	https://strokefoundation.org.au/	Yes
	Stroke Association	https://www.stroke.org.uk/	Yes
	American Heart Association	https://www.heart.org/	Yes
	American Stroke Foundation	https://americanstroke.org/	No
	Stroke Foundation NZ	https://www.stroke.org.nz/	No
	Stroke SA	https://www.stroke.org.au/	No
	World Stroke Organization	https://www.world-stroke.org/	No
	Safe Implementation of Treatments in Stroke	https://www.sitsinternational.org/	No
	Stroke Care International	https://www.strokecareinternational.org/	No
