# Supplementary material for: Nutrition Education Programs for Adults with Neurological Diseases Are Lacking: A Scoping Review
Source: Nutrients. 2022 Apr 10;14(8):1577. doi: 10.3390/nu14081577 (PMC9030740; doi:10.3390/nu14081577)
Supplement: Supplementary file 1 [file nutrients-14-01577-s001.zip › Scoping review_Nutrition education neurol_table S4.pdf]

Nutrition education programs for adults with neurological diseases are lacking: a scoping review

Table S4. Studies excluded at full text stage of screening and reasons for exclusion.

Citation	Reason for exclusion
1. Almeida CS, Stanich P, Salvioni CC, et al. Assessment and nutrition education in patients with amyotrophic lateral sclerosis. *Arquivos de Neuro-Psiquiatria* 2016; 74: 902-908. DOI: 10.1590/0004-282X20160145.	No intervention details (unclear if individualised)
2. Arshia H and Jeyaraj SS. Assessment of nutritional status and Expanded Disability Status Scale in women with multiple sclerosis in Chennai (South India) and the impact of a nutrition education program. *Multiple Sclerosis and Related Disorders* 2018; 26: 253. DOI: 10.1016/j.msard.2018.10.074.	Abstract or poster
3. Becker H, Stuifbergen A, Taxis C, et al. The use of goal attainment scaling to facilitate and assess individualized change in a wellness intervention for women with fibromyalgia syndrome. *Journal of Holistic Nursing* 2009; 27: 232-240.	Less than half of the program relates to diet
4. Block P, Skeels SE, Keys CB, et al. Shake-It-Up: Health promotion and capacity building for people with spinal cord injuries and related neurological disabilities. *Disability & Rehabilitation* 2005; 27: 185-190.	Less than half of the program relates to diet
5. Chang C-C, Wykle ML and Madigan EA. The effect of a feeding skills training program for nursing assistants who feed dementia patients in Taiwanese nursing homes. *Geriatric Nursing* 2006; 27: 229-237. DOI: 10.1016/j.gerinurse.2006.03.007.	Treatment focus (not education)
6. Gross B, Anderson EF, Busby S, et al. Using culturally sensitive education to improve adherence with anti-hypertension regimen. *Journal of Cultural Diversity* 2013; 20: 75-79.	No neurological disease/s of interest
7. Harris L, Hankey C, Jones N, et al. A cluster randomised control trial of a multi-component weight management programme for adults with intellectual disabilities and obesity. *British Journal of Nutrition* 2017; 118: 229-240. DOI: 10.1017/S0007114517001933	No neurological disease/s of interest
8. Hill VA, Vickrey BG, Cheng EM, et al. A pilot trial of a lifestyle intervention for stroke survivors: Design of healthy eating and lifestyle after stroke (HEALS). *Journal of Stroke & Cerebrovascular Diseases* 2017; 26: 2806-2813. DOI: 10.1016/j.jstrokecerebrovasdis.2017.06.058	Informative article or protocol paper
9. Ifejika NL, Noser EA, Grotta JC, et al. Swipe out Stroke: Feasibility and efficacy of using a smart-phone based mobile application to improve compliance with weight loss in obese minority stroke patients and their carers. *International Journal of Stroke* 2016; 11: 593-603. DOI: 10.1177/1747493016631557.	Informative article or protocol paper
10. Kim H and Kim O. The lifestyle modification coaching program for secondary stroke prevention. *Journal of Korean Academy of Nursing* 2013; 43: 331-340.. DOI: 10.4040/jkan.2013.43.3.331.	Full text not in English
11. Kim J-I, Lee S and Kim J-H. Effects of a web-based stroke education program on recurrence prevention behaviors among stroke patients: A pilot study. *Health Education Research* 2013; 28: 488-501.	Less than half of the program relates to diet
12. Larsen G. *Dietary outcomes from the V-STOP Stroke Program*. Dissertation. M.S., Texas Woman's University, Ann Arbor, 2012.	Cannot extract neurological disease/s of interest
13. Marck CH, De Livera AM, Brown CR, et al. Health outcomes and adherence to a healthy lifestyle after a multimodal intervention in people with multiple sclerosis: three year follow-up. *PLoS ONE* 2018; 13: e0197759.	Less than half of the program relates to diet
14. Nordvik I, Myhr KM, Nyland H, et al. Effect of dietary advice and n-3 supplementation in newly diagnosed MS patients. *Acta Neurologica Scandinavica* 2000; 102: 143-149. DOI: 10.1034/j.1600-0404.2000.102003143.x.	Supplementation trial
15. Rashvand F, Abtahi M, Moshtagh Eshgh Z, et al. Improvement in activity of daily living and fatigue in multiple sclerosis patients: the impact of nutrition education. *Nursing and Midwifery Studies* 2016; 5. DOI: 10.17795/nmsjournal32862.	No outcomes of interest
16. Riemann-Lorenz K, Eilers M, Schulz KH, et al. Multiple sclerosis and diet: systematic review, internet-based survey and pilot-testing of an evidence based patient	Abstract or poster

education programme. *Multiple Sclerosis* 2016; 22: 390. DOI: 10.1177/1352458516663081.	
17. Rimmer JH and Hedman G. A health promotion program for stroke survivors. *Topics in Stroke Rehabilitation* 1998; 5: 30-44.	Informative article or protocol paper
18. Sabour H, Javidan AN, Soltani Z, et al. The effect of behavioral intervention and nutrition education program on serum lipid profile, body weight and blood pressure in Iranian individuals with spinal cord injury: A randomized clinical trial. *Journal of Spinal Cord Medicine* 2018; 41: 28-35. DOI: 10.1080/10790268.2016.1209890.	No neurological disease/s of interest
19. Sajatovic M, Tatsuoka C, Welter E, et al. A targeted self-management approach for reducing stroke risk factors in African American men who have had a stroke or transient ischemic attack. *American Journal of Health Promotion* 2018; 32: 282-293.	Less than half of the program relates to diet
20. Sakakibara BM, Lear SA, Barr SI, et al. Development of a chronic disease management program for stroke survivors using intervention mapping: The Stroke Coach. *Archives of Physical Medicine & Rehabilitation* 2017; 98: 1195-1202.	Informative article or protocol paper
21. Suominen MH, Kivisto SM and Pitkala KH. The effects of nutrition education on professionals' practice and on the nutrition of aged residents in dementia wards. *European Journal of Clinical Nutrition* 2007; 61: 1226-1232. DOI: 10.1038/sj.ejcn.1602639.	Treatment focus (not education)
22. Suominen MH, Puranen TM, Jyvakorpi SK, et al. Nutritional guidance improves nutrient intake and quality of life, and may prevent falls in aged persons with alzheimer disease living with a spouse (NuAD Trial). *Journal of Nutrition, Health & Aging* 2015; 19: 901-907.	Individual tailored nutrition advice
23. Teuschl Y, Matz K, Firlinger B, et al. Preventive effects of multiple domain interventions on lifestyle and risk factor changes in stroke survivors: evidence from a two-year randomized trial. *International Journal of Stroke* 2017; 12: 976-984. DOI: 10.1177/1747493017702662.	Less than half of the program relates to diet
24. Wallace R, Lo J and Devine A. Tailored nutrition education in the elderly can lead to sustained dietary behaviour change. *Journal of Nutrition, Health & Aging* 2016; 20: 8-15. DOI: 10.1007/s12603-016-0669-2.	No neurological disease/s of interest
25. Weber B, Bersch-Ferreira ÂC, Torreglosa CR, et al. Implementation of a Brazilian Cardioprotective Nutritional (BALANCE) Program for improvement on quality of diet and secondary prevention of cardiovascular events: A randomized, multicenter trial. *American Heart Journal* 2019; 215: 187-197. DOI: 10.1016/j.ahj.2019.06.010.	Cannot extract neurological disease/s of interest
26. Wong YY. The effectiveness of an educational intervention on managing feeding difficulties for residents with dementia. *Dissertation Abstracts International Section A: Humanities and Social Sciences* 2018; 79.	Treatment focus (not education)
